# Starch and Glycogen Analyses: Methods and Techniques

**DOI:** 10.3390/biom10071020

**Published:** 2020-07-09

**Authors:** Henrike Brust, Slawomir Orzechowski, Joerg Fettke

**Affiliations:** 1 Leibniz Institute for Plasma Science and Technology, Institute of Biochemistry and Biology, Felix-Hausdorff-Str. 2, 17489 Greifswald, Germany; henrike.brust@inp-greifswald.de; 2 Department of Biochemistry and Microbiology, Warsaw University of Life Sciences-SGGW, ul. Nowoursynowska 159 Budynek 37 P/12B, 02-776 Warszawa, Poland; slawomir_orzechowski@sggw.edu.pl; 3 Biopolymer Analytics, Institute of Biochemistry and Biology, University of Potsdam, Karl-Liebknecht-Str. 24-25 Building 20, 14476 Potsdam-Golm, Germany

**Keywords:** starch, glycogen, analytics

## Abstract

For complex carbohydrates, such as glycogen and starch, various analytical methods and techniques exist allowing the detailed characterization of these storage carbohydrates. In this article, we give a brief overview of the most frequently used methods, techniques, and results. Furthermore, we give insights in the isolation, purification, and fragmentation of both starch and glycogen. An overview of the different structural levels of the glucans is given and the corresponding analytical techniques are discussed. Moreover, future perspectives of the analytical needs and the challenges of the currently developing scientific questions are included.

## 1. Introduction

Today, several techniques for the analysis of complex carbohydrates exist, though a single all-embracing method is lacking. All methods provide information about glycan samples, but along with this knowledge, there is unfortunately also a partial loss of specific information. As an example, very large and complex glycans can be analyzed only following partial fragmentation. Thus, a combination of several methods is required for extensive characterization of glycans. Furthermore, the use of overlapping methods is the most promising approach. Therefore, in this article, several competing techniques are presented, essentially restricted to the analytics of the complex storage glucans, starch, and glycogen. However, as starch and glycogen have very similar chemical properties but strongly different physical characteristics, this article starts with a focus on the required differences in the analytical workflow for both glucans. Following this, the different levels of analyses are summarized and discussed. However, many different conventional and modern analytical techniques exist, and thus, we constrain our discussion to the most widespread state-of-the-art methods and techniques.

## 2. Structural Organization and Differences between Starch and Glycogen

**Figure 1:**
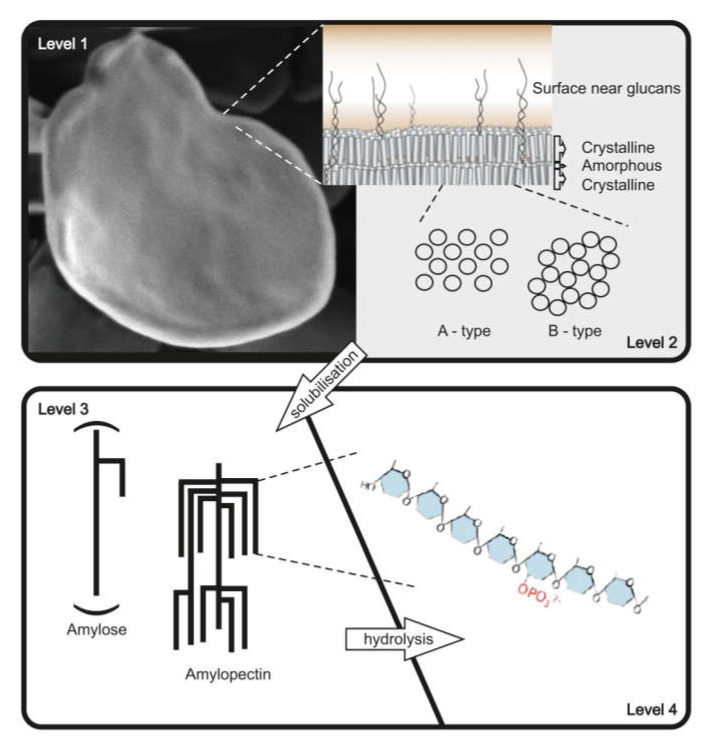
The complex hierarchical structure of starch. At least four levels can be distinguished. Level 1 represents the microscopic level of starch granules and in parts also surface properties. Level 2 reflects the inner starch structure and further information about the starch granule surface. Thus, it is necessary to distinguish between surface, crystalline, and amorphous regions. Furthermore, the crystalline regions can be organized in two major types of allomorph A and B. The level 3 represents the description of entire amylose and amylopectin molecules. Please notice, that amylose chains are much longer than amylopectin chains; this is indicated by showing only a section of amylose. The intra molecular description represents the level 4. For more information regarding starch structure, please see also [11].

Starch, which is composed of two glucose polymers, amylopectin and amylose, and glycogen serve as important reserve polysaccharides for the storage of carbon and energy in many species among Eukaryota, Bacteria, and Archaea [1,2]. The glucan polymers consist of α-D-glucosyl residues, connected via α 1,4 and α 1,6 glycosidic bonds. α 1,4 glucan chains are connected via α 1,6 linkages. While both, starch and glycogen, are chemically identical, major differences in their physicochemical properties are related to the molecular organization of glucan chains within the molecules. In starch, branching points are clustered, in contrast to glycogen, resulting in longer linear glucan chains, that can form double helices and water is excluded. Organization of double helices within amylopectin results in mainly two crystalline allomorphs (type A and B) (see also Figure 1) [3,4,5,6,7]. As a general consequence, starch and glycogen differ in their water solubility. Starch consists of branched water insoluble semi-crystalline amylopectin, and the nearly linear amylose is probably interspersed within the amorphous regions of amylopectin [8,9,10]. Glycogen, in contrast, is mostly watersoluble. Starch shows a relative high density of approximately 1.5 gcm^−3^. Consequently, the isolation methods for starch and glycogen also differ (see Section 2).

Both polymers, glycogen and starch, can be described and characterized on different structural levels, but as a consequence of the difference in complexity, the necessary levels for comprehensive characterization differ. For starch, at least four levels of structural description can be distinguished (Figure 1), whereas for glycogen, using the same structural levels, three are mostly sufficient (Table 1). Different techniques are necessary to analyze the varying structural levels (Table 1). However, a further consequence of the semi-crystalline structure of starch, in contrast to glycogen, is the necessity of solubilization. Only by solubilization of the starch granules can the structural levels 3 and 4 be analyzed.

## 3. Isolation from Tissue and Quantification of Starch and Glycogen

The generation of the homogenate is critical, as this is dependent on the tissue from which starch or glycogen is to be isolated. Transitory starch, isolated from leaves, is mostly obtained by first homogenizing frozen leaf material with mortar and pestle in liquid nitrogen following further homogenization with a blender [12,13,14] or using an all-glass homogenizer in presence of perchloric acid [15]. Isolation of storage starch is more heterogeneous as the organs and tissues differ largely. Homogenization of starch storing tissues by cutters, cryogrinder, mills, or blenders is widespread [16,17,18,19,20,21]. The extraction procedure is often combined with chemical or enzymatic treatments to remove proteins, lipids, and non-starch related carbohydrates [19,22,23]. The generation of artificial glycogen and starch species by partial destruction is to be avoided. Furthermore, an inactivation of starch endogenous enzymes (e.g., by using detergence) to prevent alterations of the glucan structure is advisable. Therefore, different adaptations are necessary for every tissue.

Following homogenization, the starch, as water insoluble particles can be easily separated from most proteins, nucleic acids, lipids, and soluble sugars by aqueous extraction and following centrifugation. Additional filtration via mesh and density gradients (e.g., Percoll) is frequently used [24,25,26]. However, thus far, it is unclear if this treatment is also accompanied by an unwanted granule size selection and/or by a loss of structural information, at least at the starch granule surface. Therefore, the data obtained regarding the starch granule surface should be critically reviewed.

The isolation of glycogen is more complex, as glycogen is watersoluble, similar to potential contaminating proteins and further metabolites. For the extraction of glycogen from mammal liver or muscles, the trichloroacetic acid (TCA)-based isolation procedures or ultracentrifugation in combination with sucrose gradients are widely used (TCA, [27,28]; sucrose, [29,30,31]). Isolation of glycogen from bacteria can be achived via sonification or a French press to disrupt the cell wall. Here, cell debris are centrifuged and the supernatant containing glycogen is precipitated with ethanol or ethanol in combination with KCl or LiCl [32,33]. In some cases, glycogen can become insoluble as e.g., in case of Lafora disease [34], and this has an impact on the further isolation, that is more similar to that of starch.

In several plant mutants affected in starch metabolism, due to the loss or reduction in starch debranching enzymes, especially isoamylases, is phytoglycogen [35,36,37,38,39]. It is characterized by its high similarity to glycogen, but its origin is from plants. Similar to glycogen, it is watersoluble, and thus, its isolation procedure is comparable. However, it can also include further isolation steps using perchloric acid or fractionation via size exclusion chromatography [38,40,41,42,43,44].

The determination of the starch or glycogen amount in a specific tissue, organ or entire living system does not inevitably include the isolation of the glucans. Thus, for starch as well as for glycogen, enzymatic and non-enzymatic procedures exist, allowing the approximate determination of the content following destruction of the biological material (see below). Iodine staining is frequently used to visualize starch and glycogen even within tissues, however false positive signals are possible as these tests are not strictly highly specific for starch or glycogen, as other glucosyl residues containing poly- and oligomers, e.g., maltodextrins, can interfere, and therefore, additional analyses are necessary for higher precision.

As an example, companies are selling starch kits (e.g., Megazyme Total Starch Assay Kit). These kits are in principle based on the enzymatic or chemical hydrolysis of starch, resulting in the exclusive formation of glucose monomers. The glucose is further enzymatically converted via glucose-6-phosphate to 6-phosphogluconolactone by hexokinase (EC 2.7.1.1) and glucose-6-phosphate dehydrogenase (EC 1.1.1.49), resulting also in a conversion of nicotinamide adenine dinucleotide phosphate (NADP^+^) into the reduced form NADPH. The formation of NADPH is then spectroscopically measured (light absorbance at 334, 340, or 365 nm). In addition, also NAD^+^ converting glucose-6-phosphate dehydrogenases can be applied forming NADH. It should be mentioned that such quantification method measures total starch comprising both components amylopectin and amylose. Information about amylose and amylopectin proportion within starch is gained by iodine staining methods and/or combination with further fractionation methods (see Section 5).

## 4. Analysis of Entire Starch Particles and Glycogen Molecules

In contrast to glycogen, the water insoluble starch granules can be easily analyzed in terms of their morphology by various microscopic methods (structure level 1, Table 1). The observed starch morphologies are typically species- and tissue- specific. The size of the starch granules isolated from different species also varies widely from below 1 µm up to several 100 µm [45,46]. For example, transitory starch granules are mostly in a range below 7 µm having a flat, discoid, or slightly round shape [47,48,49,50,51]. Starch granules from different storage organs have a bigger variability in size and shape, e.g., potato (*Solanum tuberosum* L.) starch granules are spherical with up to 100 µm in diameter. Maize starch granules are irregular-polyhedral shaped with a size range between 5 to 20 µm [45]. Wheat and barley endosperm contain two different starch granule populations, with sizes between 10 and 40 µm (A type) and below 10 µm (B type) having a discoid and spherical granule shape, respectively [52,53,54,55]. For the sake of clarity, it is to mention, that here A and B-type starch particles are not related to A and B type allomorphs, thus to the inner starch structure. Moreover, for endosperm of rice and oat also compound starch granules were described [56,57,58,59].

The morphology of starch granules can be assessed by various microscopic methods. Traditional light microscopy techniques can give some information, e.g., the detection of the Maltese cross and hilum reveals the ordered inner structure of the starch [60,61,62,63,64]. Confocal microscopy is a powerful technique for morphology analysis of starch granules *in situ* [65,66,67]. Furthermore, the staining of starch granules with pseudo-Schiff propidium iodide allows fast and easy detection of starch granules in tissues [68,69].

**Figure 2:**
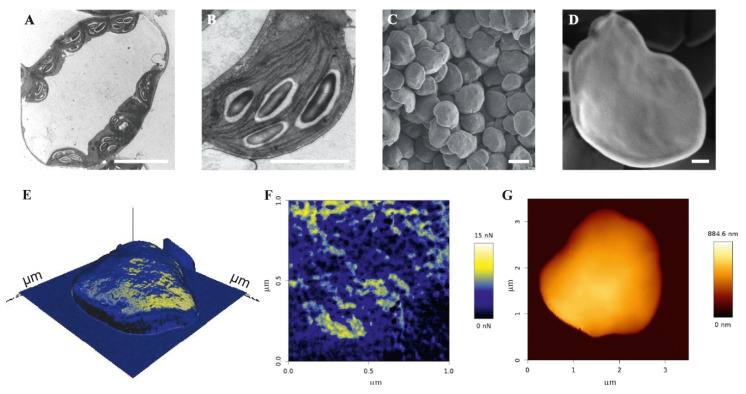
Microscopic analyses of leaf starch granules from *Arabidopsis thaliana* wild type Col-0 *in situ* and *in vitro* analyses. **A** and **B**: *in situ* analysis; Transition electron microscopy (TEM) of a mesophyll cell including chloroplast with starch granules (**A**) and of a chloroplast with starch granules (**B**). *In vitro* analysis; **C** and **D**: Scanning electron microscopy (SEM) of isolated native starch granules. The bars are equal to 10 μm (**A**), 2 μm (**B**), 1 μm (**C**), and 200 nm (**D**). **E**–**G**: *In vitro* analysis; atomic force microscopy images. **E**. Overview scan in adhesion (retract) mode. **F**. Zoom in of the adhesion (retract) scan. **G**. Height measurement scan of the same starch granule.

Today, transmission (TEM) and scanning (SEM) electron microscopic methods are mainly used (Figure 2A–D). TEM allows the analysis of starch granules inside plastids, cells, and tissues following fixation and contrasting the ultra-thin slices [50,56]. However, the information obtained for the starch granules is always limited by the section of the cut. Consequently, only two-dimensional data are collected. Using multiple sections, three-dimensional data can also be obtained, although this requires increased effort. In addition, granule shrinking can occur due to dehydration of the tissues during preparation, resulting in an underestimation of the starch granules size. SEM is used for the analysis of isolated starch granules (structure level 1, Table 1) [16,45,70,71,72]. Starch granules are often coated in vacuum with a thin layer of gold but can also be used without any coating or pre-treatment [71,73]. In principle, SEM of starch granules may also allow the detection of modifications such as phosphorylation via the energy dispersive X-ray analysis (EDX detector) or wavelength dispersive X-ray analysis (WDX). Thus, the combination of SEM with EDX/WDX detection allows to gain information about internal structure at the structural level 2. These detectors monitor the characteristic energy and intensity of elements contained in the sample and therefore allow for the detection of, e.g., phosphor compounds. However, so far, such data have not been published for starch.

Furthermore, inner structural information, such as the lamella architecture within the starch, can be obtained of intact polymers (structural level 2, Table 1) by various X-ray techniques, especially small- and wide-angle scattering (SAXS and WAXS, respectively; [74,75]) and X-ray diffraction (XRD; [72]). Atomic force microscopy (AFM) has also been applied to starch analysis [73,76,77,78,79,80,81,82,83]. AFM allows to determine the properties of starch surface structures, including starch modifications on the surface. However, due to the three-dimensional characteristics of starch granules and the resulting large altitude differences accompanied by the limitations of AFM in the z-axis, only parts of entire starch granules can be mostly analyzed (Figure 2E–G).

In addition to the morphological descriptions of starch granules, the sizes of starch granules are also of interest. Supplemental to the described microscopic methods that allow (especially by three- dimensional analyses) a relatively accurate determination of starch granule sizes and volumes (structural level 1, Table 1). Multisizer can be applied to get fast statistical information about population of granule sizes and volumes [16,84,85,86,87,88,89,90]. The electrical sensing zone method, used here, is based on the increase in electric resistance by particles in an electrical field passing an aperture or pore between two electrodes. Therefore, the electrical sensing zone method is unaffected by the particle color, shape, composition, or refractive index. Also flow cytometric analyses of starch granules have been reported [91,92,93].

As a result of microscopic methods, individual or multiple starch granules can be described, whereas the multisizer can give an overview of the size distribution of a population of starch granules. However, based on the measurement principle, the determined size of the starch granules is an approximation. In contrast to most microscopic methods the isolation of starch granules is strictly necessary for multisizer and SEM analyses. Therefore, the determined sizes of the starch granules must be critically reviewed in regard to the isolation procedure, especially considering, e.g., (partial) rupture of the starch granules and size-selective isolation. Similarly, the sizes of glycogen molecules can be directly determined, only following isolation, by separation techniques such as liquid chromatography and field flow fractionation. Coupling with multi angle laser light scattering allows for the most precise determination of the weight-average molecular weight of glycogen molecules [30,94,95,96,97]. However, also here, only a population of glycogen molecules can be described, and the accuracy is very sensitive to the isolation of glycogen.

In principle, nuclear magnetic resonance (NMR) can also be applied for starch analyses (structural level 2, Table 1). Thus, solid-phase NMR can be used for the analysis of entire starch granules [90,98,99,100,101]; however, in most cases so far, the starch is solubilized or further degraded prior to NMR analyses to allow analyses of covalent starch modification, such as phosphate esters [72,75,98,99,102,103,104,105,106,107,108]. Similarly, NMR analysis was applied to glycogen [105,109].

## 5. Solubilization and Fractionation of Starch into Amylose and Amylopectin

As out lined before, structural levels 3 and 4 (see Table 1) can only be analyzed following solubilization of the starch granules. In principle, various methods for solubilization exist.

When starch granules are heated in water, their semi-crystalline nature is gradually eliminated, resulting in structural breakdown and starch polymer dispersion in solution. This heat-induced phase transition from an ordered granular structure into a disordered state in water is known as gelatinization [110,111,112,113]. However, analyses of thermal properties allow the comparison of starches and indications for alterations in the internal starch structure, but they will not allow determination of the inner starch structure in more detail.

In addition to exclusive heat treatment of the isolated starch, additional procedures exist. All these methods can be sub-grouped into enzymatic or chemical treatments. The latter are mostly connected with a heat treatment. In connection with the further applied analytical techniques, several solvents such as dimethyl sulfoxide (DMSO) [106,114,115,116,117,118,119,120]; NaOH, KOH, urea/NaOH [121], and ZnCl_2_ [122] are used. Treatment with KOH or NaOH is the most common, as fewer limitations occur in down-stream processing.

Treatment with starch-degrading enzymes is also possible but results in a massive loss of structural information of structure levels 3 and 4 depending on the enzyme used. Therefore, it is important to distinguish between the use of enzymes for solubilization and the application of enzymes for structural analysis. The latter is of interest for both starch and glycogen. As both glucans only consist of α 1,4 and α 1,6 linkages, only enzymes that act on these linkages can be applied (see below).

Independent of the solubilization method, the time of treatment is critical as partial or total solubilization can be achieved.

Furthermore, following solubilization, isolation of the two polyglucans types, amylopectin and amylose is also possible. Several techniques are commonly in use to fractionate starch into its components, amylopectin and amylose, based on their different physicochemical properties (e.g., solubility, diffusion, hydrodynamic, and complexing properties due to degree of branching, molecular weight). Mandatory for the separation of both types of polyglucans is the solubilization of purified/extracted starch leading to the dissolution and loss of crystallinity. Storage starches are commonly dissolved using organic solvents (DMSO) or alkaline solutions (NaOH, KOH), often together with heating. In addition, use of physical methods such as autoclaving or heating via microwaves leads to swelling of the granules and destruction of the crystalline structure in aqueous solution [123,124,125]. Heating of starch in aqueous solution below the melting point of amylopectin (solubilization of amylopectin is avoided) leads to leaching of amylose [126,127,128,129]. Amylopectin is pelleted by centrifugation, while leached amylose remains in the supernatant. Efficiency of the leaching procedure is strongly dependent on the starch concentration, temperature, heating and cooling rate, and duration [130]. Moreover, the procedure takes several hours or days. In addition, an increase in fraction purity and yield is achieved when subsequently the leached amylose is precipitated [130,131,132].

The complex formation of amylose with hydrophobic substances, such as n-butanol, thymol, or a mixture of n-butanol and isoamyl alcohol, leads to precipitation of amylose that can be separated from amylopectin by centrifugation [133,134,135,136,137,138]. Amylopectin in the supernatant is recovered by ethanol or methanol precipitation [125,134,135,139]. Repetitive dissolution and precipitation steps are applied to obtain polysaccharide fractions [139,140].

The separation of amylopectin and amylose with concanavalin A is based on the ability of the lectin to bind non-reducing ends of glucans [141,142,143,144]. As the concanavalin A homotetramer has four binding sites, amylopectin molecules precipitate very efficiently, and amylose resides in the supernatant after centrifugation [144,145,146]. Megazyme International Ltd. (Wicklow, Ireland) offers a kit to separate amylose and amylopectin to measure their contents. The concanavalin A-based method is applied to a wide variety of starches from different origins such as cassava [147], common cattail [148], kiwi [149], potato [147], rice [131], tomato fruit [150], quinoa [151], and yam bean [152].

The different molar masses of amylose and amylopectin allow for separation via chromatographic methods such as size exclusion chromatography (SEC) [153]. SEC is used for the quantification of each component within starches and as a preparative method for further analysis [152,154,155,156,157].

A widely used technique to measure amylose contents in starch samples or starch fractions is based on the ability of amylose and amylopectin to bind iodine with different capacities. Binding of iodine with amylose leads to the formation of deep-blue complexes, while binding with amylopectin results in a reddish-brown color formation. Maximal absorbance of iodine bound to amylopectin is between 500–560 nm, and that for iodine bound to amylose is above 580 nm [158,159]. Absorption of the amylose–iodine complex is recorded spectrophotometrically at defined wavelength maxima between 600 and 680 nm for different starch samples (e.g., for 600 nm, [160,161,162]; for 620 nm [20,163,164,165,166,167,168]; for 625 nm [169,170]; for 635nm [23,125,171,172]; for 640 nm [19]). Absorbance of 1 mg starch in 100 mL with a defined concentration of iodine and potassium iodide at 680 nm is usually referred to as the “blue value” [173,174,175,176,177,178]. Calibration curves at defined wavelengths with defined starch amounts are used to calculate amylose contents within starch samples [163,164,166,170,171,179,180]. Recording of wavelength spectra in a range between 270–900 nm or recording of absorbances at two to three defined wavelengths are also applied to analysis of the maximum absorbance and sufficiency of iodine concentration for different starch samples and for the analysis of amylopectin and amylose, respectively [125,160,167,181]. Iodine’s affinity to amylose can also be measured by potentiometric iodine titration [111,144,155,169,182] and by amperometric iodine titration [134,170,177,183].

It should be noted that complexing of amylose with lipids influences both iodine binding capacity and the butanol precipitation procedure. Treatment and precipitation of solubilized starch with propanol [184], ethanol [171], or methanol [164,179,180] defats starch prior to further fractionation or content measurements. Thus, pre-treatment of unfractionated starch samples with alcohols has become established for estimation of apparent amylose contents by iodometric methods [111,171,185,186]. Moreover, some starches containing amylopectin with long and extra-long glucan chains (>100 glucosyl residues) reveal higher iodine binding values and can lead to overestimation of amylose contents within starch samples [187,188,189,190]. This can be overcome by fractionation of starch and separate measurements of starch components to consider the impact of amylopectin component.

## 6. Enzymatic Treatments of Starch and Glycogen

To get further intra molecular structural information (structure level 4, Table 1) it is necessary to specific fragment the polymers in oligoglucans prior to further separations (see below). Starch and glycogen contain exactly the same inter-glycosidic linkages, α 1,4 and α 1,6. Consequently, the same enzymes can be applied for the structural analysis of both polysaccharides.

Most common is the use of amylases. α-Amylases (EC 3.2.1.1) are endo-hydrolytic enzymes that cleave inner α 1,4 glycosidic linkages and consequently release maltose, maltotriose, or branched oligosaccharides. As the enzyme is unable to cleave terminal α 1,4 linkages, the release of glucose is not observed. α-Amylases comprise a wide collection of enzymes from all biological classes, such as animals, plants, fungi, and bacteria. These α-amylases have different product specificities. However, all α-amylases form a product that has the α-configuration at the anomeric carbon. β-Amylases (EC 3.2.1.2) also cleave α 1,4 glycosidic linkages but these enzymes are exo-hydrolases and hydrolyze glucans by a mechanism that create inversion of the configuration at the anomeric carbon, releasing β-maltose from the non-reducing end of a glucan chain. A third group of amylases contains the γ-amylases or amyloglucosidases (EC 3.2.1.3). These enzymes are also exo-hydrolases, cleaving α 1,4 glycosidic linkages but releasing β-glucose from the non-reducing end of a glucan chain.

In contrast, isoamylases (EC 3.2.1.68) cleave α 1,6 glycosidic linkages and therefore release linear chains from both glycogen and starch.

All the different amylases are widespread, and thus, there are enzymes with various temperature and pH optima available. Bacterial amylases have been reported that have very high temperature stability and an optimal temperature of activity around 100 °C (e.g., Wu et al., 2018).

α 1,6 glycosidic linkages can also be cleaved by glycogen-debranching enzymes mediating an indirect debranching. In a strict sense, these monomeric enzymes, found in animals and fungi, possess two enzymatic activities coded in their single polypeptide chains. One enzymatic activity is an α 1,4 glucanotransferase (EC 2.4.1.25) that transfers a linear α 1,4 glucan chain except the single glucosyl residue attached via α 1,6 glycosidic bond. The second enzymatic activity is an α 1,6 glycosidase (EC 3.2.1.33) that releases the glucosyl residue as free glucose. Despite the amylases that can be used for structural analysis of starch and glycogen, glycogen-debranching enzymes are rather unfavorable, as the resulting product is difficult to interpret. However, enzymes can also be used for total degradation of the polyglucans to glucose, as performed in the course of determining the amounts of the storage glucans [191,192]. Therefore, amyloglucosidases or mixtures of several enzymes are used.

In contrast, for the determination of the chain length distribution pattern (CLD, see below) for glycogen and starch, the selective hydrolysis of α 1,6 glycosidic linkages by a direct debranching enzyme, e.g., isoamylase is necessary. Also sequential hydrolyses in combination with phosphorylase a and/or β-amylases and/or α-amylases to get different kinds of limit-dextrins are in use to get information about structural organization of branching points and glucan chain lengths [193].

Interestingly, enzymatic treatment is also used for native starch granules that have not been solubilized. Here, specific properties of the starch granule surface can be analyzed, such as the surface near glucan chains [16,71]. Moreover, these analyses can also include various enzymes that can elongate existing glucan chains at the starch granule surface, such as starch synthases and glucan phosphorylases [16,71,194,195]. From these experiments, information about the starch surface can be found and different starches can be compared. In principle, two types of analyses can be distinguished. In the first, enzymes are used, and their catalytic action is determined, e.g., the release of glucan chains from the starch granule surface or the elongation of the surface near glucan chains. The sensitivity can be increased by including radioactive labels [13,16,71,194,195,196]. In a second type, only the binding of the enzymes at the starch granule surface is followed. This allows the user to collect information about starch surface properties when the binding characteristics of the enzymes are known (see below).

In similar experiments analyzing the native starch granule surface, enzymes can be included that do not affect α 1,4 or α 1,6 glycosidic linkages, such as glucan water dikinase (GWD; [71,104,194,197]), or phosphoglucan water dikinase (PWD; [104,194,198]). GWD and PWD are involved in the phosphorylation/dephosphorylation cycle of the transitory starch degradation [199,200]. Both enzymes introduce phosphate groups, via a dikinase reaction, into amylopectin. GWD phosphorylates the C6-OH, whereas PWD phosphorylates the C3-OH of a glucosyl residue of amylopectin (for review see [200,201]). Both phosphorylations are the only known naturally occurring covalent modifications of starch. Thus, the binding and the action of these enzymes at the starch granule surface also allow for the determination of starch properties and the differentiation of starches [71,194]. Furthermore, the use of enzymes either in combination or sequentially helps to increase the knowledge about starch surface properties [71,194].

## 7. Methods for the Characterization of Glucans Released from Starch and Glycogen

Solubilized starch and glycogen as well as released glucans following enzymatic or chemical treatment can be analyzed by various methods to determine the molar mass of the molecules as well as the size distribution and thus information of structural level 3 and 4 (Table 1) can be collected.

In some cases, a fast separation of released glucans and remaining insoluble starch granules or large remaining parts of starch and glycogen can be achieved by simple centrifugation or the usage of microfilter units. In addition, precipitation of glucans is common using increasing concentrations of methanol, ethanol or salts as described above.

Two often-used techniques are size exclusion chromatography, as mentioned previously [95,97,117,118,202,203,204,205,206,207,208,209,210,211,212,213,214] and field flow fractionation, which allow the separation of small oligoglucans up to polyglucans [94,206,215,216,217]. The advantage of the latter is the lack of potential glucan-matrix interactions that can result in unclean separation and false correlation [218,219]. It should be noted that these techniques are also used for entire amylose, amylopectin, and glycogen molecules [97,204,206,220,221,222,223,224].

**Figure 3:**
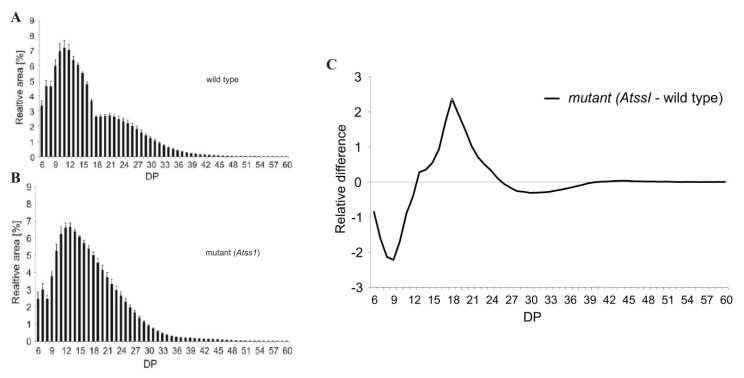
Typical chain length distribution (CLD) profiles of starches from *Arabidopsis thaliana* analyzed by HPAEC-PAD. (**A**) and (**B**) CLD profiles from wild type and *Atss1* mutant. Plants were grown in a 12 h light/12 h dark cycle and leaves were harvested at the end of the light period. The isolated starches were digested with isoamylase to degrade the α 1,6 linked branching points within the amylopectin. (**C**) Difference plots were obtained by subtraction CLD profile of wild type from mutant CLD. Differences of mean values are presented. DP—degree of polymerization.

Today, several faster and more sensible modern methods exist. Most common is the analysis of enzymatically processed starch and glycogen products with capillary electrophoreses (CE) or high performance anion exchange chromatography (HPAEC). The maximum degree of polymerization (DP) limit for both methods is approximately 70. Both methods are differentially compatible with the detection of glucans following separation. In CE, the glucans are mostly detected by fluorescence (laser induced fluorescence; LIF), and thus, coupling of the carbohydrates via the reducing end is necessary with a fluorescent dye (e.g., APTS, ANTS). In contrast, no derivatization of the glucans is needed following separation of the glucans by HPAEC; instead, amperometric detection, especially pulsed amperometric detection (PAD) is common. A typical CLD profile of debranched transitory starch from *Arabidopsis thaliana* displays a polymodal distribution that is diminished in mutant plants lacking the major soluble starch synthase isoform (Figure 3) [51,225]. Absolute quantification of glucan chains separated and detected by HPAEC-PAD is limited as suitable standards are missing and sensitivity of PAD signal is decreasing with increasing glucan chain lengths. However, amperometric detection is fast and cost-effective and gives reliable information about different kinds of wild type and mutant starches [51,187,225,226,227,228,229,230]. However, the obtained CLD profiles, following enzymatic debranching (e.g., by isoamylase) do not allow to explain the branching pattern within the molecule, as it only gives an average of the included glucan chains.

Smaller glucans released from starch or glycogen can also be analyzed by mass spectrometry, mostly using matrix assistant laser desorption and ionization (MALDI) mass spectrometry with 2,5-Dihydroxybenzoic acid (DHB) as matrix [207,231,232]. MALDI mass spectrometry is in principle also possible in a high-throughput process using spotting devices; however, the collected data are mostly semi-quantitative, and therefore, it is comparatively less effective than CE-LIF. However, for analysis of *in vitro* or *in vivo* modifications of starches, starch- and glycogen-like molecules and glycogen, MALDI mass spectrometry is the method of choice [197,233,234]. In addition to the detection of entire phosphoglucans, the internal position of the phosphate group can be determined. This comprises the phosphorylated carbon of a glucosyl residue (C6 or C3-OH group) as well as the position of glucosyl residue within in the glucan chain.

## 8. Protein Binding Analyses

In addition to the carbohydrate-related analyses of starch and glycogen, protein analytics of both carbon stores can also be performed. For starch and glycogen, binding and integration of proteins have been reported. Thus, for glycogen, the binding of glycogenin, glycogen synthase, glycogen phosphorylase, glycogen debranching enzyme, laforin as well as kinases and phosphatases were reported [235,236,237,238]. In the case of starch, at least two alternatives can be distinguished: The binding of proteins at the starch granule surface or the integration of proteins inside the granule. Several examples and proteomic analyses exist for both [239,240,241,242,243,244,245]. The most prominent is the integration of the granule-bound starch synthases (GBBS), thought to be responsible for the generation of amylose [9,246,247,248], into starch granules from several species (amaranth [249]; barley [250,251]; maize [252,253]; pea [25,254]; potato [159,255]; rice [256]; and wheat [257,258]). Here, the proteins cannot be washed away under any conditions. The proteins can only be released if the starch is solubilized or chemically or enzymatically degraded. Therefore, it is evident that these proteins are not related to contamination or artefacts. The opposite is the case for proteins bound to the starch surface. In addition to several identified proteins related to starch metabolism, e.g., GWD [12,13,241], PWD [13,241], PHS1/PHO1 [239,240,241,242,243], ESV1 [194,241,259], FLO6 [260], PTST1 [241,261], LESV [241,259], LSV2 [241], SEX4 [241], debranching enzyme isoforms [239,240,241,245], starch synthase isoforms [21,239,241,242,243,262,263,264], starch branching enzyme isoforms [21,239,241,242,243,262,264,265,266,267,268], and proteins not directly involved in starch metabolism were also reported [21,239,241,245].

To date, it is unclear whether some integrated or bound proteins are also important for the overall structural properties of the starch granules.

Besides analysis of catalytic actions of enzymes at the starch granule surface and its resulting glucan products (see Section 6) also the binding of proteins or enzymes to native starch granules allows the collection of further information about the starch structural characteristics *in vitro*. Such experiments can also be extended by pre-treatment of the starch via, for instance, hydrolytic digestion by enzymes [66]. Moreover, protein carbohydrate analysis with soluble polyglucans (e.g., solubilized starches and starch fractions) typical methods, such as NMR, isothermal titration calorimetry, fluorescence spectroscopy, surface plasmon resonance, micro scale thermophoresis, and biolayer interferometry can be applied. Binding of proteins to starch granules in *vivo* can be analyzed by using transgenic plants expressing proteins labelled with a fluorescent group, such as e.g., GFP, YFP, or mCherry [261,269,270,271]. Fluorescence microscopy allows the determination of the distribution of the proteins within the plastids and at the starch granule (surface). Thus, a clustered distribution can be easily distinguished from a randomly even allocation of the binding protein. Furthermore, by combining of several differently labelled proteins, co-localization can be monitored. However, it should be mentioned, that due to the altered (over)expression of these proteins within plants a generation of artefacts must be considered.

## 9. Conclusions and Future Perspectives

Over the past few decades various carbohydrate analysis techniques have been established, and subsequently, the starch and glycogen fields have benefitted. Thus, more and more detailed information about starch and glycogen has been obtained. A covalent modification, the phosphorylation of glycogen and starch, was identified, and various mutants with different starch morphologies, altered inner structures, and modified surface properties have been reported.

However, many open questions remain. For example, for both starch and glycogen, covalent modification by phosphorylation has been detected. However, to date, the molecular order of this phosphorylation event is unclear. Thus, e.g., the exact position of the phosphorylation, i.e., the glucosyl residue in the glucan chain, the distance to branching points, the distance between two phosphorylation events, and the physical or chemical background are obscure. Similar to the modification of starch and glycogen, the order at the surface of both polyglucans is far from being resolved. More and more results point to specific characteristics of the surface that influence the interaction with proteins, and thus, the changing surface is critical for biological function. Thus, for biological and biochemical analyses, the surface is the focus of the research, as almost all interactions with proteins occur here. This includes the synthesis as well as the degradation of new glycosidic linkages. Furthermore, the results of the action of enzymes and proteins during synthesis at the surface presuppose the created inner structure. In addition, both the actions of enzymes and proteins during synthesis and degradation presuppose the available surface. In this dynamic molecular world, the currently available methods are limited. This limitation comprises, a time component as well as an individual component. Thus, for many analyses, at least the isolation of glycogen or starch is needed, which reduces the time resolution of possible analyses. Additionally, it can also alter parameters of the analyte. Furthermore, as most of the analyses cannot be performed with a single starch or glycogen molecule, due to the low amount, almost all data to date are averages, limiting your insight. Depending on the analytic question and method, this averaging can include different physiological or metabolic states of the same cell but can also include analytes from different cells, tissues or even organs. Thus, especially small alterations can be underestimated.

Similarly, in regard to the analyses of the inner structure, nearly no individual starch or glycogen molecule has been analyzed to date, limiting our knowledge about possible natural variance. Interestingly, an analysis of a single giant starch granule from orchid was reported [272].

However, analyze of characteristics such as the size and shape and the resulting properties for applications when huge amounts of the polyglucans are necessary, especially starch, can be more accurate, faster, and obtained more cheaply. Therefore, analytic needs differ largely among the various scientific and industrial sectors. However, gaining more insight into individual molecular structures will also be helpful for crude industrial applications. Furthermore, in computational science, particularly modelling, it has become more and more important to develop new ideas about the exact properties of starch and glycogen.

## Figures and Tables

**Table 1:** Levels of molecular and supra molecular structure organization.

Level of Structural Description	Preparation	Main Analytical Methods	Glucans
**Level 1 Microscopic level** - size - shape - morphology - surface structures	native, partially hydrolyzed, or mechanically destroyed starch granules isolated or in tissue	TEM, SEM, AFM, light microscopy, multisizer	starch; (crystallized glycogen) *
**Level 2 Internal structures** - conformation and helical structures of the glucan chains - crystallinity - arrangement of glucan chains within the granules	non-invasive, isolation of granules partially necessary	XRD, solid NMR, EDX, WDX, SAXS, WAXS, XRD	native and solubilized starch; glycogen
**Level 3 Whole molecules** - size of molecules	solubilization of starch granules is required	SEC/GPC, FFF	amylopectin; amylose; solubilized starch; glycogen
**Level 4 Intra molecular** - glucan chains - branching frequency - CLD distribution - chemical modifications	partial and sequential hydrolysis, specific hydrolysis of α1,4 or α 1,6 glycosidic linkages	HPAEC-PAD, CE, SEC, NMR, MS	amylopectin; amylose; solubilized starch; glycogen

TEM—transmission electron microscopy; SEM—scanning electron microscopy; AFM—atomic force microscopy; XRD—X-ray diffraction; NMR—nuclear magnetic resonance spectroscopy; EDX—energy dispersive X-ray spectroscopy; WDX—wavelength-dispersive X-ray spectroscopy; SAXS—small-angel X-ray scattering; WAXS—wide-angel X-ray scattering; XRD—X-ray powder diffraction; SEC—size exclusion chromatography; GPC—gel permeation chromatography; FFF—field flow fractionation; HPAEC-PAD—high performance anion exchange chromatography with pulsed amperometric detection; MS—mass spectrometry; *—very limited application.
